# Multiorgan Drug Action of Levosimendan in Critical Illnesses

**DOI:** 10.1155/2019/9731467

**Published:** 2019-09-19

**Authors:** Jian Pan, Yun-Mei Yang, Jian-Yong Zhu, Yuan-Qiang Lu

**Affiliations:** ^1^Department of Emergency Medicine, The First Affiliated Hospital, School of Medicine, Zhejiang University, Hangzhou 310003, China; ^2^Department of Geriatric Medicine, The First Affiliated Hospital, School of Medicine, Zhejiang University, Hangzhou 310003, China

## Abstract

Cardiotonic drugs mainly include digitalis, catecholamines, phosphodiesterase inhibitors, and calcium sensitizers, which have been successively discovered and applied in clinical practice. However, there are only a few new drugs available in this field, and the selection is very limited. Digitalis, catecholamines, and phosphodiesterase inhibitors increase myocardial contractility by increasing intracellular concentrations of cyclic adenosine monophosphate (cAMP) and Ca^2+^, and this increase in intracellular calcium ion concentration enhances myocardial oxygen consumption and causes arrhythmia. For these reasons, the research focus on positive inotropic agents has shifted from calcium mobilization to calcium sensitization. Intracellular calcium sensitizers are more effective and safer drugs because they do not increase the intracellular concentration of calcium ions. However, only three calcium sensitizers have been fully developed and used in the past three decades. One of these drugs, levosimendan, has multiple molecular targets and exerts its pharmacological effects by not only increasing myocardial contractility, but also enhancing respiratory muscle function and liver and kidney protection, and it is useful for patients with severe sepsis and septic shock. Recently, more than 60 randomized controlled clinical trials of levosimendan have been reported; however, these clinical trials have occasionally shown different findings. This article reviews the research progress of levosimendan in critical illnesses in recent years.

## 1. Introduction

**Figure 1:**
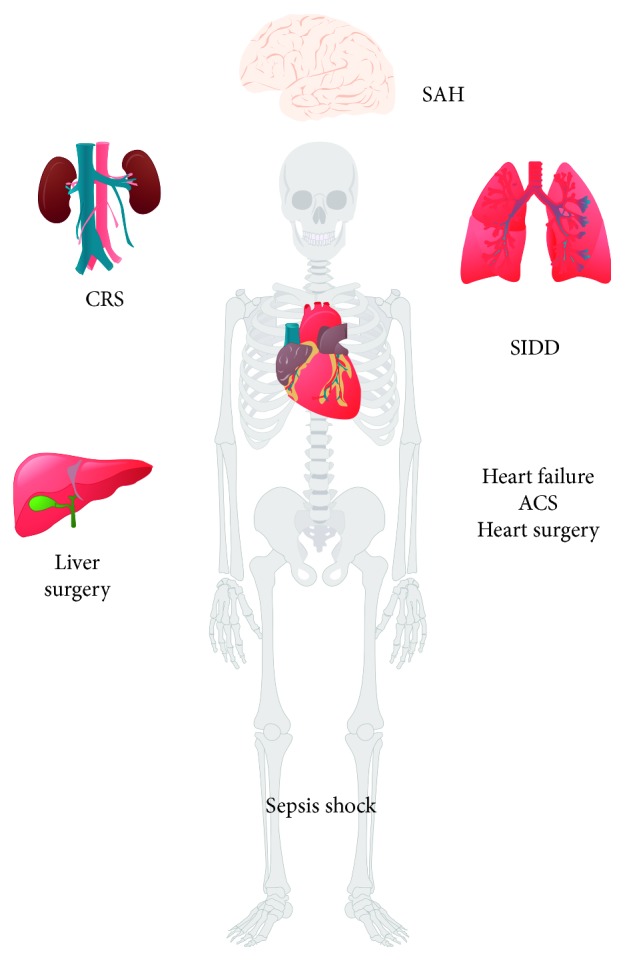
Multiorgan drug action of levosimendan. SAH, subarachnoid hemorrhage; CRS, cardiorenal syndrome; SIDD, sepsis-induced diaphragm dysfunction; ACS, acute coronary syndrome.

Preliminary pharmacological studies of levosimendan and its metabolites have shown that levosimendan has an intracellular calcium-sensitizing effect. In addition, its activation of adenosine triphosphate- (ATP-) sensitive potassium channels on vascular smooth muscle membranes produces profound vasodilation. Furthermore, its activation of mitochondrial ATP-sensitive potassium channels exerts a cardioprotective effect and causes selective phosphodiesterase inhibition. These data suggest that the cardiovascular effects of levosimendan are not only merely based on drug-receptor interactions, but also based on its unique and beneficial functions, such as energy metabolism, antioxidative stress, and neurohormonal function, which contrast other types of positive inotropic-vascular drugs. Other studies have shown that levosimendan enhances respiratory muscle function, improves liver and kidney function and subarachnoid hemorrhage, and changes the prognoses of patients with severe sepsis and septic shock (Figure 1).

### 1.1. Levosimendan: Mechanism of Action

Levosimendan is a calcium sensitizer and exerts its inotropic effect via binding to the Ca++ saturated troponin C of the myocardial thin filament. This action results in stabilization of the Ca-bound conformation of troponin, thereby prolonging the actin-myosin interaction without altering cross-bridge cycling [1]. Although levosimendan inhibits phosphodiesterase III, its inotropic effect seems to depend almost entirely on its calcium-sensitizing properties [2]. Consequently, compared with other inotropic agents, levosimendan does not increase calcium flux into the cell, and this could explain why levosimendan may actually improve diastolic function and does not increase oxygen consumption in the myocardium [3]. Because levosimendan may increase myocardial oxygen supply through coronary vasodilation, so it is recommended for use in patients with acute heart failure and acute coronary syndrome [4–6]. Levosimendan causes vasodilation through its effect on K^+^ channels in high plasma concentrations [7]. Levosimendan also induces vasodilation in other organs, including the myocardium, lungs [8], liver, and kidney. As a result, organ perfusion is improved despite a slight drop in blood pressure in some patients. The clinical consequences of levosimendan-related tissue perfusion improvement should be evaluated taking in consideration concurrent improvement in cardiac output. In addition to its inotropic and vasodilator effects, levosimendan has several other important effects, including increase in diaphragm contractility [9, 10], anti-inflammatory effect [11–13], and antiapoptotic effect [14, 15], and affects platelet function [16–18]. In addition, levosimendan stimulated iNOS expression and nitric oxide (NO) production [19, 20]. But these mechanisms are not clear and need further study.

### 1.2. Levosimendan and Heart Failure

We identified four noteworthy randomized controlled trials of acute decompensated heart failure. These trials mainly covered patients with acute decompensated heart failure and heart failure after myocardial infarction. The severe low-output heart failure study (the LIDO study) compared heart failure patients with less than 35% left ventricular ejection fraction (LVEF) that were given levosimendan treatments with patients given dobutamine treatments. Results of the LIDO study showed that levosimendan had a significant advantage in stabilizing hemodynamics and 180-day mortality of the patients [21]. In evaluating the safety and efficacy of levosimendan in patients with left ventricular failure complicating acute myocardial infarction (RUSSLAN trial), continuous intravenous injection of low-dose levosimendan in patients with heart failure after acute myocardial infarction did not cause adverse reactions, such as hypotension and myocardial ischemia, and significantly reduced the 14-day and 180-day mortality rates compared to patients in the placebo group [22]. SURVIVE trial (levosimendan vs. dobutamine for patients with acute decompensated heart failure) compared responses of patients with acute decompensated heart failure under levosimendan and dobutamine treatments; the results showed no significant difference in 180-day mortality between the two groups. The levosimendan treatment significantly reduced the brain-type natriuretic peptide (BNP) levels in patients after 24 h and continued this beneficial effect for five days. No significant differences in the all-cause mortality, survival time, discharge rate, and 24 h dyspnea were found 31 days posttreatment. Patients in the dobutamine treatment group had significant increases in the incidence of acute heart failure, while patients in the levosimendan treatment group had a higher incidence of adverse reactions, such as atrial fibrillation, hypokalemia, and headache. Nevertheless, the subsequent in-depth analysis on the Finnish subgroup of patients showed a significant difference in mortality between levosimendan and dobutamine treatments. The Finnish patients who underwent the levosimendan treatment had higher beta-blocker usage, and a higher proportion of patients with acute myocardial infarction were compared with the Finnish patients who underwent the dobutamine treatment [23].

REVIVE trial (effect of levosimendan on the short-term clinical course of patients with acute decompensated heart failure) compared the treatment responses of decompensated heart failure patients after levosimendan treatment with those of the placebo group, and the results showed that the levosimendan treatment significantly reduced the BNP levels in patients at 6 h, 24 h, and five days posttreatment. Furthermore, the clinical deterioration rate of patients who received the levosimendan treatment significantly reduced. In brief, although the levosimendan group had more cardiovascular adverse effects than the placebo group, levosimendan rapidly and persistently relieved clinical symptoms [24].

These four major randomized controlled trials of levosimendan had various tested subjects, observation indicators, and study endpoints and focused on different interpretations of results [25]. However, overall, levosimendan is found to improve hemodynamics and mortality in patients more significantly than dobutamine. Levosimendan also improves hemodynamics in patients when compared with the placebo group (Table 1).

Importantly, among the four clinical trials, the RUSSLAN trial focused on the treatment of heart failure after acute myocardial infarction. Since then, several studies on levosimendan used for acute coronary syndrome have focused on the evaluation of its safety and efficacy in patients with acute coronary syndrome accompanied by acute heart failure and even cardiogenic shock [22, 26]. Based on these studies, the consensus recommendations are as follows: (1) patients benefit from the levosimendan treatment, which enhances myocardial contractility, stabilizes hemodynamics, improves target organ perfusion, and reduces the hospital readmission rate; (2) compared to other vasoconstrictors, levosimendan has a lower incidence of adverse reactions, such as hypotension, headache, atrial fibrillation, hypokalemia, and tachycardia; (3) the application of levosimendan depends on the heart failure severity, mean arterial pressure, heart rate, and infarct size; and (4) levosimendan can be used in combination with other vasoconstrictors under continuous electrocardiogram (ECG) monitoring.

### 1.3. Levosimendan and Respiratory Muscle Function

Mechanical ventilation is an important method of life support for patients with critical illnesses. Approximately 40% of patients in intensive care units receive mechanical ventilation, and approximately 25% of the patients encounter difficulties when weaning themselves off mechanical ventilation [27]. The diaphragm, the most important respiratory muscle, is responsible for 60%–80% of breathing work. Diaphragmatic dysfunction caused by sepsis can lead to prolonged mechanical ventilation and increased complications and mortality in patients. Diaphragmatic dysfunction caused by sepsis has been a hot topic in modern critical care medicine and a blind spot in clinical treatment. Active treatment of the primary disease cannot easily improve its clinical outcomes in the short term, and other methods, such as adjusting ventilator parameters or sequentially using invasive and noninvasive therapies, are not ideal [28]. Promotion of diaphragmatic cell regeneration and reduction of apoptosis may be suitable treatments [29].

The diaphragm is a special skeletal muscle that can easily become fatigued. If the body is under stress and the diaphragm has long-term passive contraction, the diaphragm's metabolism will be high and it will be subjected to intense stress. Thus, the diaphragm will be prone to increasing the hydrolyzed protease expression, structural damage of the muscle fibers, and disuse atrophy [30, 31]. Within three days of mechanical ventilation, disuse atrophy of diaphragmatic fibers occurs in the diaphragm muscle, expressions of caspases 1, 3, 4, 8, and 11 increase due to inflammation, and apoptosis and pyroptosis take place [27, 32, 33].

One study by van Hees et al. showed that intravenous injection of levosimendan enhances the contraction of diaphragmatic muscle fibers by increasing intracellular calcium sensitivity, which provides strong theoretical support for calcium sensitizer treatment in patients with respiratory muscle dysfunction accompanied by chronic obstructive pulmonary disease [9].

A study by Schellekens et al. showed that levosimendan did not affect inflammation of the diaphragm caused by mechanical ventilation and endotoxemia in a mouse model [34]. A follow-up study by Doorduin et al. comparing the intracellular venous injection of levosimendan to placebos in healthy individuals showed that levosimendan significantly enhances the action potential and muscle contractility of the diaphragmatic nerves [10]. In a study by Sterba et al., intravenous injection of levosimendan facilitates the withdrawal of mechanical ventilation in patients with low cardiac output by increasing the LVEF, and oxygenation index; however, it does not improve other indicators, such as respiratory muscle strength or renal function [35]. In a randomized double-blind study by Gordon et al, the median time spent on the ventilator in adult patients with severe sepsis and levosimendan treatment was two days more than that in the control group, with no statistically significant difference [36]. A study of challenges in withdrawing mechanical ventilation which compared the difference between levosimendan and dobutamine treatments in chronic obstructive pulmonary disease patients showed a greater reduction in the pulmonary wedge pressure in the levosimendan treatment group [37]. In general, a few studies have evaluated the effect of levosimendan on the function of respiratory muscles. More basic and clinical experiments should be carried out to investigate whether levosimendan can help patients overcome the challenges associated with withdrawing from mechanical ventilation.

### 1.4. Levosimendan and Liver and Kidney Functions

Studies on the effects of levosimendan on liver and kidney functions are still very few. Existing reports have shown that levosimendan may have a protective effect on liver and kidney functions, and this effect may be derived from the drug, which acts directly on the liver and kidney and is unlikely to be related to the improvement of cardiac function. A study by Oktar et al. in a rat model of acute-heart-failure-induced liver failure showed that levosimendan prevents hepatic ischemic-reperfusion injury (IRI) in rats through histological examinations and specific immunohistochemistry [38]. Studies by Onody et al. and Grossini et al. showed that levosimendan prevents hepatic IRI in rats after portal vein embolization, and the specific mechanism may be related to the mitochondrial ATP-sensitive potassium channels. Thus, levosimendan pretreatment in patients with partial liver resection may have a certain protective effect on the liver [39, 40]. A study by Brunner et al. also showed that levosimendan reduces apoptosis in human hepatocytes after ischemia-reperfusion injury [41].

Acute and chronic heart failure combined with renal insufficiency, also known as type 1 and type 2 cardiorenal syndromes, respectively, are very common in clinical practice. General treatments for these syndromes involve using diuretics to reduce cardiac preload, positive inotropic agents to increase myocardial contractility, and renal replacement therapy. Fedele et al. believed that levosimendan may have potential benefits for patients with acute heart and kidney syndromes accompanied by hypotension. However, its specific mechanism still needs further study for validation [42]. The European Heart Association recommends using levosimendan in patients with advanced heart failure or heart failure accompanied by renal insufficiency to improve the renal outcomes by dilating renal blood vessels and increasing renal blood perfusion and to improve the prognosis of patients [43, 44]. However, no significant effect of levosimendan on renal function was reported in either of the two randomized controlled trials of REVIVE I and II [24]. Several animal studies have shown that levosimendan improves cardiac function as well as reduces the pressure of renal arteries, ultimately increasing the renal blood flow [45–47]. Some studies have shown that levosimendan improves renal function in patients with congestive heart failure [48], especially heart failure patients with low ejection fraction, which may be related to the selective dilation of renal arteries and veins and increased renal perfusion [49]. Levosimendan also increases renal blood flow and improves the glomerular filtration rate in patients after cardiac surgery [50–52]. Limited studies of levosimendan effects on septic shock have shown that levosimendan increases creatinine clearance in plasma [53]. The Levosimendan for the Prevention of Acute Organ Dysfunction in Sepsis (LeoPARDS) trial showed that the incidence of new acute kidney event, new requirement for renal replacement therapy, and sustained renal failure (stage 2 or 3 acute kidney injury) at day 28 and the duration of renal replacement therapy did not differ significantly between the levosimendan group and the control group. The duration of renal replacement therapy was two days shorter in the levosimendan group than in the control group, although the difference was not statistically significant [36].

### 1.5. Levosimendan and Severe Sepsis and Septic Shock

Sepsis has been increasingly recognized as causing organ damage due to a microbial invasion of the body, leading to systemic inflammatory dysfunction and immune response. An increasing number of studies have shown that lung, kidney, heart, and skeletal muscles may become damaged by sepsis. Use of levosimendan in severe sepsis and septic shock is a hot research topic in patients with critical illnesses. More than 10 randomized controlled trials of this type are available, trials with positive results accounting for the majority [54–61] and one trial showing neutral results [36]. A 2016 study by Gordon et al. in *The New England Journal of Medicine*, with the largest number of tested subjects to date, demonstrated that levosimendan significantly reduces mortality in patients relative to normal controls. Although this study has somewhat neutral results, researchers still have great hope for levosimendan in the treatment of sepsis. In a previous study, levosimendan did not improve the function of vital organs compared to the standard controls and it required a larger dose of norepinephrine to maintain mean arterial pressure and resulted in a higher incidence of supraventricular tachycardia. However, this study has some significant data imbalances and design insufficiencies [62]. For example, the inclusion rate of heart failure patients after myocardial infarction in this study is low. The second important shortcoming of this study is the absence of a control group; the only comparison made is between the standard group and the same group added with the levosimendan treatment.

**Figure 2:**
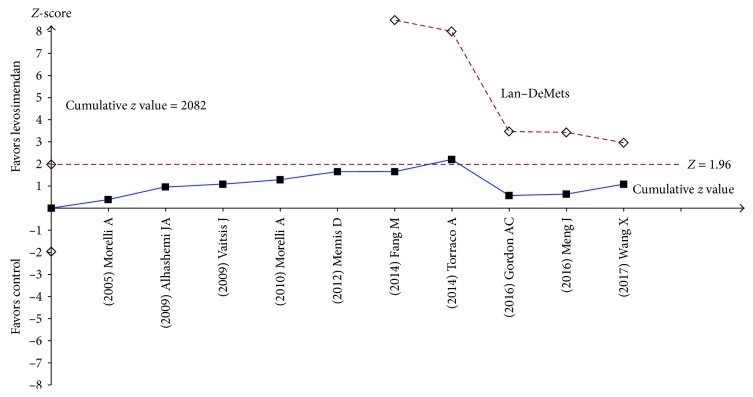
TSA: the optimal information size of 2082 patients for detection of the plausible treatment effect of levosimendan in sepsis and the Lan–DeMets sequential monitoring boundary constructed by the optimal information size did not cross (reproduced from Chang et al., [65]).

Comparison of some recent meta-analyses has shown little difference among the selected randomized controlled studies. However, their conclusions are completely different. In 2015, a meta-analysis of seven large randomized controlled trials by Zangrillo et al. showed that patients with severe sepsis and septic shock that were given levosimendan treatment had significantly lower mortality than those taking dobutamine [63]. However, subsequent meta-analyses have shown negative results. For example, Bhattacharjee et al. selected seven randomized controlled trials and concluded that levosimendan treatment had no beneficial effects compared to dobutamine treatment [64]. In 2018, a meta-analysis of 10 recent randomized controlled trials conducted by Chang et al. showed that the levosimendan group had no beneficial results compared to the dobutamine or control groups. The sequential analysis of the total number of cases (TSA) showed that the number of cases involved was too small to obtain reliable findings (Figure 2) [65]. These randomized controlled trials had different population inclusion criteria, drug concentrations for treatment, treatment regimens for controls, and study endpoints. As a result, the homogeneity of these meta-analyses was not strong. Therefore, the lack of consistency in the results of these different meta-analyses is reasonable.

### 1.6. Levosimendan and Cardiogenic Shock

Cardiogenic shock complicates approximately 5% of myocardial infarctions with a high hospital mortality rate approaching 27%–51% [66–70]. In cardiogenic shock complicating myocardial infarction, early revascularization of the occluded vessel by percutaneous coronary intervention is the first-line strategy. A supportive approach is to give mechanical support, such as intra-aortic balloon pump (IABP), ventricular assist device (VAD), and extracorporeal membrane oxygenation (ECMO). We often choose dobutamine as the inotropic agent for the support of cardiogenic shock. Because levosimendan can cause hypotension in some patients during continuous injection, there are some worries about the use of levosimendan in patients with cardiogenic shock. Recent studies on the use of levosimendan in cardiogenic shock have shown that levosimendan is associated with the improvement in hemodynamics and cardiac function, but with no significant improvement in survival [71].

A retrospective cohort study about patients hospitalized in ICU undergoing venoarterial (VA) ECMO was conducted in a French university hospital from 2010 to 2017; results suggested that levosimendan can exert a beneficial effect on VA-ECMO weaning in ICU patients [61]. And it may reduce the need for high-dose inotropes in another study [72]. There is no more evidence on levosimendan therapy for acute viral myocarditis [73, 74] and postpartum cardiomyopathy [75, 76] in humans to date.

## 2. Conclusion and Future Perspective

To date, more than 60 clinical trials on levosimendan have been reported, including more than ten thousand patients. However, the results of those trials have been inconsistent, which creates great confusion for physicians trying to select positive inotropic agents for patients with acute and chronic heart failure or severely infected patients with myocardial stunning. Despite the development of this medicine and the completed clinical trials in this field, the exact role of levosimendan in respiratory muscle, liver, and kidney function, and in the neuroendocrine aspect, still remains unclear (Table 2). This review article summarizes the findings of major studies on levosimendan and concludes that levosimendan can significantly improve the clinical symptoms of heart failure in patients. However, this conclusion has not been confirmed in any large randomized controlled trials [77]. The beneficial effects of levosimendan outlined above usually appear within 24 h and include significantly reducing the NT-BNP levels within five posttreatment days, improving the results of cardiac ultrasound and invasive hemodynamics, and improving hemodynamics without increasing myocardial oxygen consumption in patients. These effects can last for approximately one week and are not affected by beta-blockers and other cardiovascular drugs used by patients. Although some recent large randomized controlled trials, i.e., REVIVE, SURVIVE, and LeoPARDS, have produced neutral results, no more adverse outcomes are observed after levosimendan treatment despite those adverse reactions, such as benign arrhythmia and controlled hypotension. Therefore, further randomized controlled trials are necessary to validate the role of levosimendan. For the liver and kidney function of specific populations, these limited studies have suggested a protective effect of levosimendan, but the underlying mechanism remains to be verified. Given the variations of levosimendan effects on the function of diaphragmatic muscles and other respiratory muscles in basic and clinical studies, further studies are needed to identify the specific mechanism and the relationship with blockades of certain cell signaling pathways to reduce the number of pyroptotic diaphragmatic muscle cells.

## Figures and Tables

**Table 1:** Narrative summaries of main controlled trials of levosimendan in acute heart failure.

Study	Eligible patients	Control	Outcome
LIDO	Low-output heart failure (EF < 0.35, CO < 0.25, and PCWP > 15 mmHg)	Levosimendan vs. dobutamine	Hemodynamic performance and mortality at 31 days and 180 days
RUSSLAN	Left ventricular failure complicating acute myocardial infarction	Levosimendan vs. placebo	Mortality at 14 days and 180 days
SURVIVE	Acute decompensated heart failure (EF < 0.30)	Levosimendan vs. dobutamine	Mortality at 180 days or affect any secondary clinical outcomes
REVIVE I	Acute decompensated heart failure (EF < 0.35)	Levosimendan vs. placebo	Symptomatic benefits
REVIVE II	Acute decompensated heart failure (EF < 0.35)	Levosimendan vs. placebo	BNP declined in the levosimendan group

**Table 2:** Details of the use of levosimendan in critical illnesses.

Acute decompensated heart failure (ADHF)	Is recommended/is indicated
ADHF complicating acute myocardial infarction	
Acute coronary syndrome.	
Septic shock	May be considered
Cardiogenic shock	
Pulmonary hypertension and right ventricular dysfunction	
Heart surgery	
Weaning from ventilator	Need more research
Sepsis-induced diaphragm dysfunction	
Weaning from extracorporeal membrane oxygenation	
Cardiorenal syndrome	
Liver surgery	
Subarachnoid hemorrhage	
